# Burden of Disease Measured by Disability-Adjusted Life Years and a Disease Forecasting Time Series Model of Scrub Typhus in Laiwu, China

**DOI:** 10.1371/journal.pntd.0003420

**Published:** 2015-01-08

**Authors:** Li-Ping Yang, Si-Yuan Liang, Xian-Jun Wang, Xiu-Jun Li, Yan-Ling Wu, Wei Ma

**Affiliations:** 1 Department of Epidemiology and Health Statistics, School of Public Health, Shandong University, Jinan, Shandong, People's Republic of China; 2 Shandong Center for Disease Control and Prevention, Jinan, Shandong, People's Republic of China; Institut Pasteur, France

## Abstract

**Background:**

Laiwu District is recognized as a hyper-endemic region for scrub typhus in Shandong Province, but the seriousness of this problem has been neglected in public health circles.

**Methodology/Principal Findings:**

A disability-adjusted life years (DALYs) approach was adopted to measure the burden of scrub typhus in Laiwu, China during the period 2006 to 2012. A multiple seasonal autoregressive integrated moving average model (SARIMA) was used to identify the most suitable forecasting model for scrub typhus in Laiwu. Results showed that the disease burden of scrub typhus is increasing yearly in Laiwu, and which is higher in females than males. For both females and males, DALY rates were highest for the 60–69 age group. Of all the SARIMA models tested, the SARIMA(2,1,0)(0,1,0)_12_ model was the best fit for scrub typhus cases in Laiwu. Human infections occurred mainly in autumn with peaks in October.

**Conclusions/Significance:**

Females, especially those of 60 to 69 years of age, were at highest risk of developing scrub typhus in Laiwu, China. The SARIMA (2,1,0)(0,1,0)_12_ model was the best fit forecasting model for scrub typhus in Laiwu, China. These data are useful for developing public health education and intervention programs to reduce disease.

## Author Summary

Scrub typhus, also known as tsutsugamushi disease, is a zoonosis transmitted by chigger bites (larval trombiculid mites) and the pathogen *Orientia tsutsugamushi (O. tsutsugamushi)*, a Gram-negative obligate intracellular bacterium. It is distributed widely in the Pacific regions of Asia, and the islands of the western Pacific and Indian Oceans. People with outdoor activities that involve contact with grasses or shrubs are at highest risk. Scrub typhus has existed in Southern China for thousands of years, but it has been noted to spread from the South to the North of China in recent decades. Though this research we studied the disease burden of scrub typhus with disability-adjusted life years (DALYs), and developed a forecasting time series model for human clinical disease in Laiwu, China. Results demonstrated that the disease burden of scrub typhus was increasing year by year in Laiwu, and it was higher in females than males. Moreover, DALY rates in females and males were highest for persons in the 60–69 years age group. Of all the seasonal autoregressive integrated moving average (SARIMA) models tested, the SARIMA(2,1,0)(0,1,0)_12_ model was the best fit for scrub typhus cases in Laiwu. The disease occurred mainly in autumn, with a peak in October.

## Introduction

Scrub typhus, also known as tsutsugamushi disease, is a zoonosis transmitted by chigger bites (larval trombiculid mites) and infection with *Orientia tsutsugamushi* (*O. tsutsugamushi*), a Gram-negative obligate intracellular bacterium. Globally, scrub typhus is distributed widely in the Pacific region of Asia. It is prevalent in a triangle from Northern Japan and far-eastern Russia in the north, to Northern Australia in the south, to Pakistan and Afghanistan in the west, and also involves islands of the western Pacific and Indian Oceans [1]–[3].

Scrub typhus has existed in China for thousands of years. Before 1986, the disease was found only in Southern China, with epidemics occurring mainly in summer [4]. Beginning in 1986, scrub typhus was also reported in areas of Northern China, such as Shandong Province, Tianjin City, Heilongjiang Province, Shanxi Province and Hebei Province [5]. Clinical infections in Northern China seemed to occur mainly in autumn and winter [4].

Shandong Province is most noted to the one of the most serious foci for scrub typhus in Northern China [5]. In 1986, the first outbreak of scrub typhus occurred in Linyi District, followed other outbreaks in Jinan in 1988, in Jining in 1996, in Yantai in 1997, in Weifang and in Tai′an in 2000 [6]. From 2006 to 2012, a total of 2337 scrub typhus cases were notified in the Diseases Reporting Information System of Shandong Center for Disease Control and Prevention. At present, 13 of 17 districts have reported scrub typhus cases in Shandong Province where Laiwu is the district of the highest scrub typhus incidence.

In 1999, the earliest cases of scrub typhus were documented in Laiwu District [7]. Since 2006, scrub typhus cases have been included in the Notifiable Infectious Diseases System managed by Shandong Center for Disease Control and Prevention. From 2006 to 2012, 308 cases were detected in Laiwu (134 males and 174 females). The average annual incidence of scrub typhus in Laiwu (3.59/100,000) was approximately ten times of that in entire Shandong Province (0.35/100,000). However, researchers and health authorities have more often focused their attentions to Linyi, the initial focus of scrub typhus in Shandong, where the annual incidence was 0.89/100,000. As a new focus of scrub typhus in Northern China, Laiwu (117°19′-117°58′E,36°02′-36°33′N) lies in the center of Shandong Province, an important geographical position in Shandong. The population of Laiwu was 1,226,393.

Incidence, prevalence, duration and mortality indicators were most frequently used to estimate the burden of disease [8]. Of available indexes, we highlight disability-adjusted life years (DALYs) to measure the disease burden of scrub typhus in Laiwu, China. The DALYs metric was jointly developed by the World Bank, Harvard School of Public Health and the World Health Organization (WHO) for the Global Burden of Disease and Injury Study(GBD) [9]. The European Centre for Disease Prevention and Control (ECDC) had adopted an incidence- and pathogen-based DALYs approach to measure the Burden of Communicable Diseases in Europe Project (BCoDE) across European Member States [10]–[12].

DALY, a summary metric of population health, is often used to identify health gaps by measuring the state of a population's health compared to a normative goal which is for individuals to live the standard life expectancy in full health. One DALY means one-year loss of ‘healthy’ life. It integrates disease-specific mortality, morbidity and severity together, and quantifies morbidity associated with different clinical outcomes by assigning disability weights on a scale between 0 and 1, where by 0 means no morbidity and 1 means death [13]. DALYs and DALY rates (DALYs per 100,000 population or DALYs per 1000 population) [8], [14], are often used to compare disease burden of the same disease among regions, areas, countries, districts [15], or disease burden of different diseases in the same place [16]. Moreover, DALYs and DALY rate could be used to identify high risk population for targeted interventions or intervention prioritization. In this research, DALYs and DALY rate were adopted to evaluate the disease burden of scrub typhus in Laiwu, China.

Epidemic modeling and forecasting is more recognized as an essential tool in preventing and controlling infectious disease. Cases of scrub typhus in Laiwu, China seem to have considerable variation during the period 2006 to 2012. A seasonal time series autoregressive integrated moving average (SARIMA) modeling introduced by Box and Jenkins [17] is most useful in examining data for seasonal or periodic fluctuations that recur with about the same intensity each year. SARIMA models have been successfully applied for forecasting economic, marketing, and social problems. While this model has the advantage of accurate forecasting over short periods, it has a limitation that at least 50 observations are needed [17]. In this study, we sought to identify the most suitable SARIMA model we could use to predict scrub typhus cases in Laiwu over time.

By combined the results of disease burden measured by DALYs and DALY rate with a SARIMA forecasting model of scrub typhus, we hoped to identify high-risk populations and interventions or intervention prioritization of scrub typhus. The research could help health authorities to prevent and control of scrub typhus efficiently.

## Materials and Methods

### Data of disease and population

The dataset of the human scrub typhus cases in Laiwu, China from 2006 to 2012 was obtained from the Diseases Reporting Information System of Shandong Center for Disease Control and Prevention. The notification system recorded the detailed information for the scrub typhus cases, including gender, age, dates of symptom onset and diagnosis, and recovery or death. The symptom onset date was used in this study, and it was thought to be more useful than the date of diagnosis or the date of notification. The cases recorded were the anonymized in this study. Population data for Laiwu was obtained from the Laiwu Statistical Bureau and stratified by age group and gender [18].

### Calculation of DALYs and DALY rate

**Figure 1:**
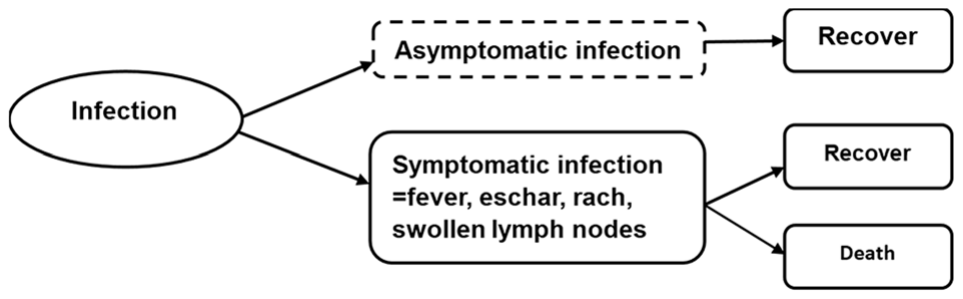
The Outcome tree for *Orientia tsutsugamushi*.

In this research, we adopted the incidence- and pathogen-based DALYs approach [10]–[13] to estimate the disease burden of scrub typhus in Laiwu, China. This approach has been previously used in the BcoDE project by ECDC. Fig. 1 shows a disease outcome tree for *Orientia tsutsugamushi* infection. All cases of scrub typhus in this study recovered for their illness.

DALYs were the sum of years of life lost due to premature death (YLLs) and years lived with disability (YLDs) [19]. According to the outcome tree, YLDs were calculated for each health outcome (l) by multiplying the number of incident cases (n) with the disability weight (w) for a specific health outcome (l), and the duration of the disabling condition (t) [see equation (1)]. All input parameters in both YLDs and YLLs formula were chosen to be age (a) and sex (s) dependent when such information was available, where a stands for age at infection and ã for age at onset of a condition or death [11], [12].
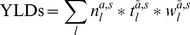
(1)


To estimate the YLLs for those health outcomes (*l*) that can lead to death, the number of fatal cases (d) for a specific health outcome (i) for an infection acquired at age (a) is multiplied by the remaining life expectancy (e) at age ã [see equation (2)].
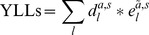
(2)


In this research, the Coale and Demeny West Level 26 Life Table adopted in many disease burden studies was used in the calculation of YLLs to assure comparability to other disease burden assessments [20]. Life expectancy for males and females at birth were set at 80 and 82.5 years, respectively. The average durations of scrub typhus in different age groups and gender were achieved by DisMod II software developed for the calculation of GBD [21]. DisMod II is powered by two basic inputs: the make-up of a region's population by gender and age, and the overall mortality rate for each demographic group. This model stratifies the relationships between a set of indicators relevant by age and gender: incidence, prevalence, remission, mortality, duration, case fatality, and RR mortality (the relative risk on total mortality) [22]. It requires powering with a minimum of three of these variables (by age groups and gender) and these three variables allow the prediction of the others [22]. Comparing the internal epidemiological consistency of estimates of incidences, prevalence, duration and mortality, we found that when inputting the variables were incidence, remission (100%), and case fatality of scrub typhus clarified by age and gender, the outputs fitted well. Thus, the average durations for scrub typhus in gender and different age groups were obtained (Table 1).

**Table 1:** The duration (year) of scrub typhus in different age groups and gender in Laiwu, China.

Gender	Age (years old)							
	0–4	5–14	15–29	30–44	45–59	60–69	70–79	80+
Male	0.9990	0.9996	0.9992	0.9975?	0.9926	0.9769	0.9367	0.8042
Female	0.9990	0.9998	0.9994	0.9988	0.9959	0.9832	0.9557	0.9061

No paper has previously reported the disability weight of scrub typhus, including “*Global Burden of Disease 2004 Update: Disability Weights for Diseases and Conditions*,” the widely adopted document about disability weights of disease [13], [16], [23]. Scrub typhus and dengue fever have so many similarities in duration, high-risk population, signs and symptoms, pathogenesis and prognosis [1], [24]–[26] that disability weight of dengue fever (0.197) [13] was referred in the research of scrub typhus.

According to BCoDE-project, these raw incidence data should be corrected for underestimation by pathogen-specific multiplication factors (MF), representing either correction for underestimation in one step, or separate correction for under-ascertainment and underreporting in two steps [12], [27]. The overall extent of underestimation can be explained by two major effects represented by under-ascertainment and under-reporting [27]. Since one person infected with *Orientia tsutsugamushi* will demonstrate acute symptoms, such as fever, rash, eschar and swollen lymph nodes, the person is likely to visit a doctor, and due to physical awareness, scrub typhus is relatively easy to diagnosis in Laiwu, China. Moreover, as a notifiable disease in Laiwu, scrub typhus must be reported to the Diseases Reporting Information System of Shandong Center for Disease Control and Prevention. So, under-ascertainment and under-reporting of scrub typhus in Laiwu was thought to be minimal and not included in the research. We used “1” as the MF value of scrub typhus in Laiwu, China in the research.

### SARIMA model

Many time series data contain seasonal periodic components. To deal with seasonality, a general multiplicative SARIMA model was extended from the ARIMA model (Box et al) [28]. This building process of the SARIMA (p,d,q)(P,D,Q)s model was designed to take advantage of associations in the sequentially lagged relationships which usually exist in periodically collected data. Seven main parameters are selected when fitting a SARIMA model: p, the order of process autoregression; d, the order of difference; q, the order of process moving average; P, D and Q, the corresponding seasonal orders; s, the length of seasonal period. If d is nonzero, a general differencing can be used to remove trend. If D is not zero, seasonal differencing can be used to remove seasonality. In order to construct and validate the model, the database of scrub typhus was verified by dividing the data file into two data sets, i.e. the data between January and December 2006–2011 and data between January and December 2012.

The original series of scrub typhus cases was not a stable time series, so it was necessary to make it stable by differential. After general difference of 1 order, followed by seasonal difference of 1 order and length of seasonal period was 12, the series was satisfied with stability. Thus d = 1, D = 1, S = 12. Then the order of autoregression and moving average were identified using autocorrelation function (ACF) and partial autocorrelation function (PACF) of the differenced series. The most suitable model was selected on the basis of Normalized Bayesian Information Criteria (BIC) [29] and Ljung-Box test. Lower values of Normalized BIC and Ljung-Box test (higher significant) were preferable. Furthermore, Ljung-Box test was performed to test if ACF of the residuals at different lag times were significantly different from zero, where no different from zero was expected [30]. Compare the predicted values in 2012 using the most suitable SARIMA model with the number of scrub typhus cases notified in 2012 to validate the forecasting ability of the model. The analyses were carried out with STATA version 12.0 (Stata Corporation, College Station, USA).

## Results

### DALYs, DALY rate and incidence of scrub typhus in Laiwu, China

No cases of death were reported in Laiwu from 2006 to 2012, so DALYs of scrub typhus in Laiwu was equal to YLDs. From 2006 to 2012, DALYs of scrub typhus were 5, 10, 10, 5, 8,10 and 13, respectively. The average annual DALYs of scrub typhus was 9, and DALYs were higher in females (5) than males (4) (Table 2).

**Table 2:** DALYs of scrub typhus in different age groups and gender in Laiwu, China (2006–2012).

Year	Gender	Age(years)								Total
		0–4	5–14	15–29	30–44	45–59	60–69	70–79	80+	
2006	M*	0	0	0	0	1	0	0	0	2
	F*	0	0	0	1	2	0	0	0	3
	B*	0	0	0	1	3	1	0	0	4
2007	M*	0	1	0	0	2	0	0	0	4
	F*	0	0	0	1	2	2	1	0	6
	B*	1	1	0	1	4	2	1	0	10
2008	M*	1	1	0	1	2	0	0	0	5
	F*	1	0	0	1	1	1	0	0	5
	B*	1	1	1	2	3	1	1	0	10
2009	M*	1	1	0	0	1	0	0	0	3
	F*	0	0	0	0	0	0	1	0	3
	B*	1	1	0	1	1	1	1	0	5
2010	M*	0	0	0	1	1	1	0	0	4
	F*	1	0	0	0	1	1	1	0	4
	B*	1	0	0	1	2	2	1	0	8
2011	M*	0	0	0	1	1	1	1	0	4
	F*	0	0	1	1	1	2	1	0	6
	B*	0	1	1	2	2	3	1	0	10
2012	M*	0	0	0	1	2	2	1	0	6
	F*	0	0	1	1	3	1	1	0	7
	B*	0	0	1	2	5	3	2	0	13
Mean	M*	0	0	0	1	1	1	0	0	4
	F*	0	0	0	1	1	1	1	0	5
	B*	1	1	0	1	3	2	1	0	9

* M, males; F, females; B, both males and females.

**Figure 2:**
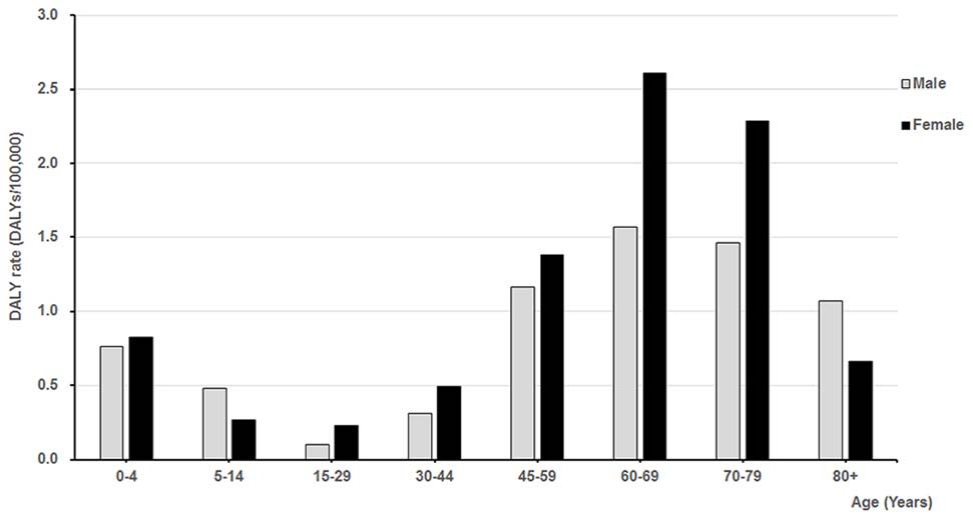
The breakdown of average DALY rate of scrub typhus in different age groups and gender in Laiwu, China (2006–2012). Scrub typhus was an acute illness. YLLs were zero. No sequelae.

DALY rates (DALYs/100,000) of scrub typhus were 0.3663, 0.7933, 0.8106, 0.4437, 0.6179, 0.7883 and 1.0618 from 2006 to 2012, respectively. The average annual DALY rate was 0.6974 DALYs/100,000. The DALY rate was higher among females (0.8019 DALYs/1 00,000) than among males (0.5961DALYs/100,000). Moreover, DALY rates in females and males were both highest for the 60–69 years age group (Table 3). Fig. 2 shows the breakdown of average DALY rates of scrub typhus in different age groups and gender in Laiwu. Since all cases of scrub typhus were cured in Laiwu, no sequelae were recorded.

**Table 3:** DALY rate (DALYs/100,000) of scrub typhus in different age groups and gender in Laiwu, China (2006–2012).

Year	Gender	Age(years)								Total
		0–4	5–14	15–29	30–44	45–59	60–69	70–79	80+	
2006	M*	0.0000	0.0000	0.0000	0.0000	0.8696	0.9135	0.9418	0.0000	0.2486
	F*	0.0000	0.0000	0.1464	0.5581	1.4550	0.4793	0.0000	0.0000	0.4877
	B*	0.0000	0.0000	0.0722	0.2737	1.1562	0.7011	0.4194	0.0000	0.3663
2007	M*	0.4863	1.1923	0.0000	0.1073	1.5655	0.9135	0.9033	0.0000	0.5943
	F*	1.0486	0.2637	0.1464	0.5581	1.8182	3.8496	2.2917	2.3098	0.9987
	B*	0.7569	0.7513	0.0722	0.3284	1.6892	2.3501	1.6734	1.4937	0.7933
2008	M*	1.4588	0.7154	0.1426	0.5366	1.3910	0.4593	1.8836	4.0058	0.7482
	F*	2.0950	0.2637	0.2930	0.7812	1.2733	2.4003	0.7510	0.0000	0.8750
	B*	1.7650	0.5009	0.2167	0.6565	1.3334	1.4090	1.2554	1.4153	0.8106
2009	M*	1.4588	0.7154	0.0000	0.1072	0.8703	0.9186	0.9033	0.0000	0.4690
	F*	1.0486	0.2637	0.0000	0.2232	0.3642	0.9624	3.0620	0.0000	0.4176
	B*	1.2613	0.5009	0.0000	0.1641	0.6225	0.9400	2.1006	0.0000	0.4437
2010	M*	0.9725	0.4769	0.1425	0.3217	0.6963	2.7406	0.0000	0.0000	0.5634
	F*	1.5728	0.0000	0.0000	0.2232	1.2727	2.3967	3.0233	0.0000	0.6742
	B*	1.2614	0.2504	0.0722	0.2734	0.9785	2.5723	1.6769	0.0000	0.6179
2011	M*	0.4863	0.2385	0.1426	0.5362	0.6944	1.3728	2.7869	3.4995	0.5830
	F*	0.0000	0.5274	0.5858	0.5582	0.9098	5.2840	3.0814	0.0000	1.0001
	B*	0.2522	0.3757	0.3612	0.5470	0.7999	3.2865	2.9503	1.2364	0.7883
2012	M*	0.4863	0.0000	0.2851	0.5363	2.0869	3.6642	2.8254	0.0000	0.9666
	F*	0.0000	0.5274	0.4393	0.5582	2.5470	2.8948	3.8131	2.3098	1.1602
	B*	0.2522	0.2505	0.3612	0.5470	2.3122	3.2877	3.3732	1.4937	1.0618
Mean	M*	0.7641	0.4769	0.1018	0.3065	1.1677	1.5689	1.4634	1.0721	0.5961
	F*	0.8236	0.2637	0.2301	0.4943	1.3772	2.6095	2.2888	0.6599	0.8019
	B*	0.7928	0.3756	0.1651	0.3986	1.2703	2.0781	1.9212	0.8056	0.6974

*M, males; F, females; B, both males and females.

The annual incidence (/100,000) of scrub typhus was 1.88, 4.08, 4.16, 2.28, 3.18, 4.08, and 5.46 from 2006 to 2012, respectively. The average annual incidence was 3.59/100,000. There was no statistical difference between incidence of scrub typhus in males and females each year (2006–2012)(*P*>0.05). Related *x* ^2^ and *P* values were showed in Table 4. But, the average annual incidence in females (4.11/100,000)was higher than males (3.07/100,000) (*x* ^2^ *= *5.850, *P* = 0.016). The average annual incidence was the highestin 60–69 age group (Table 4).

**Table 4:** Incidence (/100,000) of scrub typhus in different age groups and gender in Laiwu, China (2006–2012).

Year	Gender	Age(years)								Total	*x* ^2^ (*P*)
		0–4	5–14	15–29	30–44	45–59	60–69	70–79	80+		
2006	M*	0.0000	0.0000	0.0000	0.0000	4.4469	4.7556	5.0467	0.0000	1.2844	2.357(0.125)
	F*	0.0000	0.0000	0.7438	2.8362	7.4170	2.4818	0.0000	0.0000	2.4854	
	B*	0.0000	0.0000	0.3669	1.3909	5.9011	3.6430	2.2476	0.0000	1.8754	
2007	M*	2.4708	6.0545	0.0000	0.5459	8.0044	4.7556	5.0467	0.0000	3.0504	3.272(0.070)
	F*	5.3264	1.3388	0.7438	2.8362	9.2712	19.8546	12.1568	12.9400	5.1364	
	B*	3.8450	3.8150	0.3669	1.6691	8.6247	12.1434	8.9903	8.3682	4.0770	
2008	M*	7.4123	3.6327	0.7242	2.7294	7.1151	2.3778	10.0934	23.6855	3.8532	0.284(0.594)
	F*	10.6527	1.3388	1.4877	3.9707	6.4899	12.4091	4.0523	0.0000	4.4737	
	B*	8.9718	2.5433	1.1008	3.3381	6.8090	7.2861	6.7427	8.3682	4.1585	
2009	M*	7.4123	3.6327	0.0000	0.5459	4.4469	4.7556	5.0467	0.0000	2.4082	0.087(0.768)
	F*	5.3264	1.3388	0.0000	1.1345	1.8542	4.9636	16.2091	0.0000	2.1540	
	B*	6.4084	2.5433	0.0000	0.8345	3.1775	4.8574	11.2378	0.0000	2.2831	
2010	M*	4.9415	2.4218	0.7242	1.6376	3.5575	14.2667	0.0000	0.0000	2.8899	0.335(0.563)
	F*	7.9896	0.0000	0.0000	1.1345	6.4899	12.4091	16.2091	0.0000	3.4795	
	B*	6.4084	1.2717	0.3669	1.3909	4.9932	13.3578	8.9903	0.0000	3.1801	
2011	M*	2.4708	1.2109	0.7242	2.7294	3.5575	7.1333	15.1400	23.6855	3.0504	3.272(0.070)
	F*	0.0000	2.6777	2.9754	2.8362	4.6356	27.3000	16.2091	0.0000	5.1364	
	B*	1.2817	1.9075	1.8347	2.7818	4.0854	17.0008	15.7330	8.3682	4.0770	
2012	M*	2.4708	0.0000	1.4484	2.7294	10.6726	19.0223	15.1400	0.0000	4.9770	0.548(0.459)
	F*	0.0000	2.6777	2.2315	2.8362	12.9797	14.8909	20.2614	12.9400	5.9649	
	B*	1.2817	1.2717	1.8347	2.7818	11.8022	17.0008	17.9806	8.3682	5.4632	
Mean	M*	3.8826	2.4218	0.5173	1.5597	5.9716	8.1524	7.9305	6.7673	3.0734	5.850(0.016)
	F*	4.1850	1.3388	1.1689	2.5120	7.0196	13.4727	12.1568	3.6971	4.1186	
	B*	4.0281	1.9075	0.8387	2.0267	6.4847	10.7556	10.2746	4.7818	3.5878	

*M, males; F, females; B, both males and females.

The trend of DALY rates of scrub typhus was consistent with incidences from 2006 to 2012 in Laiwu, China. Since 2009, DALY rates and the incidences were both increasing year by year (Table 3, 4). Fig. 2 showed that in 60–69 age group, the DALY rate in females was sharply higher than males.

### SARIMA model

**Figure 3:**
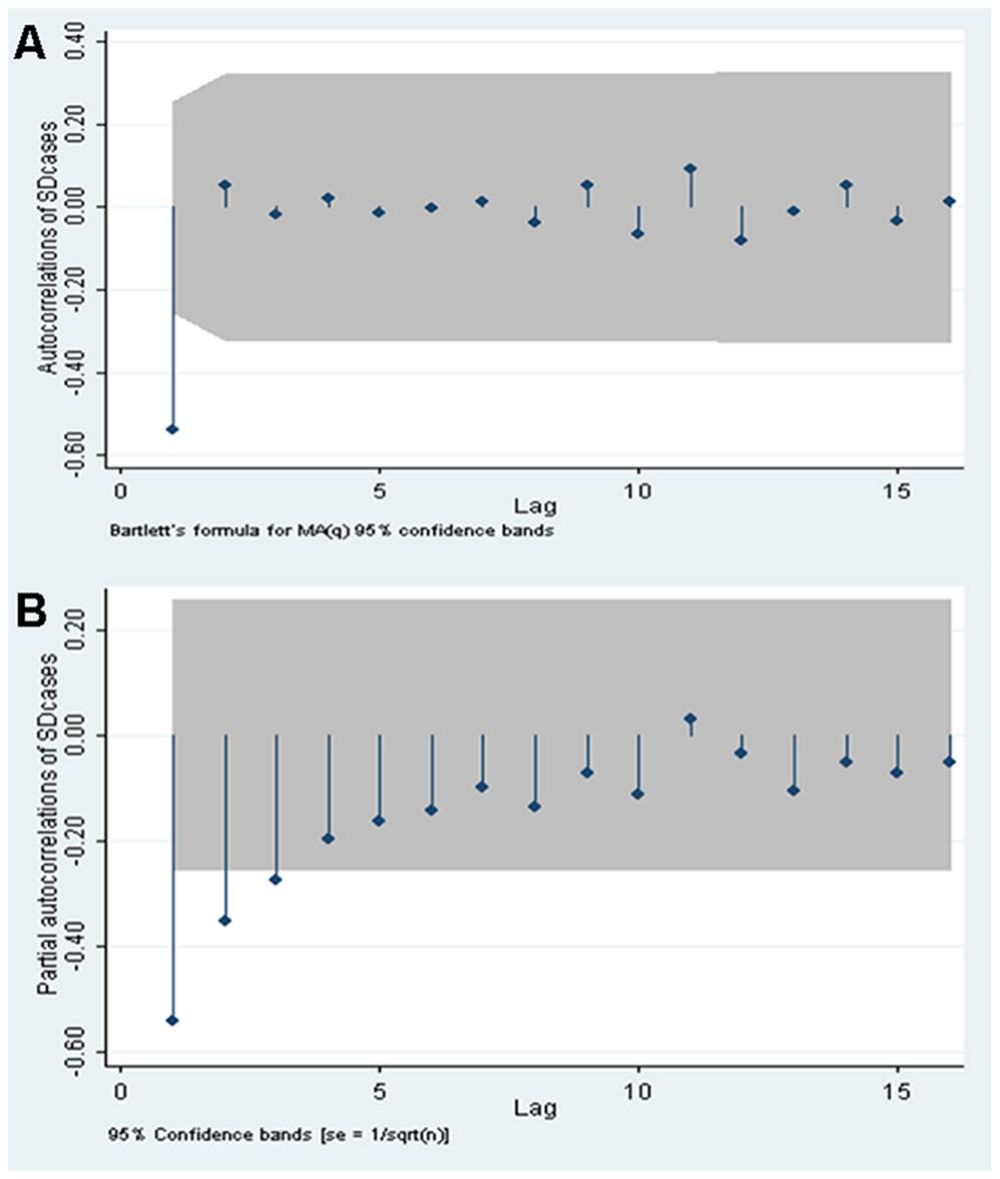
The autocorrelation function and partial autocorrelation function of differenced time series of scrub typhus cases in Laiwu, China. A, Autocorrelation function; B, Partial autocorrelation function.

The series of notified cases was a non-stationary series. Therefore, by taking 1 order general difference, followed by 1 order seasonal difference and length of seasonal period was 12, the time series of scrub typhus cases was corrected into stationary series. Fig. 3 shows the autocorrelation function (ACF) and partial autocorrelation function (PACF) of scrub typhus cases in Laiwu, China after differencing. Based on the distribution characteristic, we conducted several models, SARIMA(2,1,0)(0,1,1)_12_, SARIMA(1,1,0)(1,1,1)_12_, SARIMA(0,1,0)(2,1,1)_12_, SARIMA(2,1,0)(0,1,0)_12_, SARIMA(1,1,0)(1,1,0)_12_, SARIMA(0,1,0)(2,1,0)_12_, SARIMA(2,1,1)(0,1,0)_12_ and SARIMA(2,1,1)(0,1,0)_12_. Of all the models tested, the SARIMA(2,1,0)(0,1,0)_12_ model was the best fit for the data (Table 5). Moreover, the Ljung-Box test suggested that the ACF of residuals for the model at different lag times was not significantly different from zero, i.e. the residuals of the SARIMA(2,1,0)(0,1,0)_12_ model was satisfied with white noise. The stationary residuals provided the evidence that the SARIMA(2,1,0)(0,1,0)_12_ model was adequate. All the coefficients of the SARIMA(2,1,0)(0,1,0)_12_model were significant (Table 5, 6). The equation of the SARIMA was 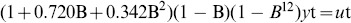.

**Table 5:** Comparisons of the tested SARIMA models for scrub typhus cases in Laiwu, China.

Model	Ljing-Box Q Statistics(*P*)	BIC*	Parameters(*P*)
SARIMA(2,1,0)(0,1,1)_12_	9.219(0.866)	3.723	AR, lag1(<0.001)
			MA, lag 2(0.013)
			MA Seasonal, lag 1(0.383)
SARIMA(1,1,0)(1,1,1)_12_	15.656(0.405)	3.912	AR, lag1(<0.001)
			AR Seasonal, lag1(0.283)
			MA Seasonal, lag 1(0.912)
SARIMA(0,1,0)(2,1,1)_12_	28.743(0.017)	4.083	AR Seasonal, lag1(<0.001)
			AR Seasonal, lag2(<0.001)
			MA Seasonal, lag 1(0.897)
SARIMA(2,1,0)(0,1,0)_12_	7.325(0.966)	3.764	AR, lag1(<0.001)
			AR, lag2(<0.001)
SARIMA(1,1,0)(1,1,0)_12_	9.676(0.883)	3.888	AR, lag1(<0.001)
			AR Seasonal, lag1(0.226)
SARIMA(0,1,0)(2,1,0)_12_	23.342(0.105)	3.990	AR Seasonal, lag1(0.001)
			AR Seasonal, lag2(<0.001)
SARIMA(2,1,1)(0,1,0)_12_	1.056(1.000)	3.638	AR, lag1(0.766)
			AR, lag2(0.823)
			MA,lag1(0.009)
SARIMA(2,1,1)(0,1,0)_12_	0.679(1.000)	3.634	AR, lag1(0.771)
			MA,lag1(0.945)
			AR Seasonal, lag1(0.348)
SARIMA(0,1,1)(2,1,0)_12_	4.918(0.993)	3.319	MA,lag1(0.981)
			AR Seasonal, lag1(<0.001)
			AR Seasonal, lag2(<0.001)

*BIC, Bayesian Information Criteria.

**Table 6:** Parameters estimated by a SARIMA(2,1,0)(0,1,0)_12_ model for scrub typhus cases in Laiwu, China.

	Coefficient	Std. err.	*P*	[95% Conf. interval]
Constant	0.023	0.411	0.060	[−0.784,0.829]
AR1	−0.720	0.072	<0.001	[−0.862,−0.578]
AR2	−0.342	0.087	<0.001	[−0.513,−0.172]

**Figure 4:**
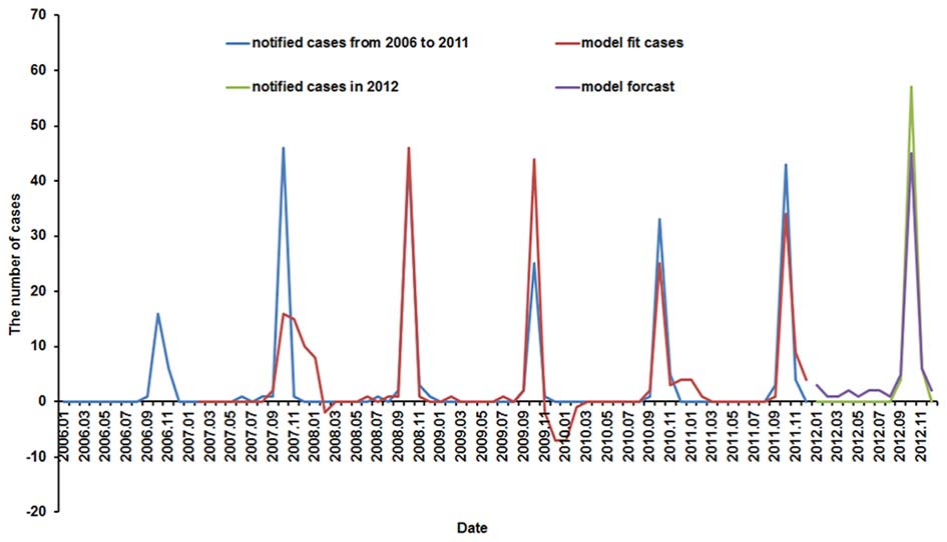
Notified cases, model fit cases (2006–2011), predictive cases (2012) of scrub typhus by SARIMA(2,1,0)(0,1,0)_12_ model.

All the number of notified cases from Jan 2012 to Dec 2012 were in the 95% confidence interval of the forecasting values by the SARIMA(2,1,0)(0,1,0)_12_ model. The model was used to predict values from January to December 2012 for validation. The notified cases and fitted cases by the best fitted SARIMA model from 2006 to 2011, and the actual cases and predicted cases from January to December 2012, were illustrated in Fig. 4. It showed that the predicted values could follow the upturn and downturn of the observed series reasonably well. In addition, the fitted values appeared some negative values, which was a common case with the series with too many zeros as observed values in the series of scrub typhus cases. The SARIMA(2,1,0)(0,1,0)_12_ model showed that the prevailing disease occurred mainly in autumn and peaked in October.

## Discussion

Incidence is often used as one measure index of disease frequency, and since its calculation only concerns the cases number and basic population data, it must be standardized by some standard population when compared between different areas or different times. However, DALYs consider both survival time and life quality caused by a certain disease and its calculation requires age and sex dependent incident cases, fatal cases, severity of disease and life expectancy. Therefore, DALYs can demonstrate the threat of some disease to the whole population, and it is more objective and comprehensive than incidence [23]. In addition, standardization issues have been covered inherently in the calculation of DALYs [8], [14]–[16], [31], [32], such as different age groups and gender are assigned with different life expectancies and different diseases are assigned with different disability weights. So, DALYs and DALY rate could be compared among areas or diseases directly. From 2006 to 2012, the average annual DALYs of scrub typhus was 9, and DALY rate was 0.6974 DALYs/100,000 in Laiwu District. The average annual DALYs and DALY rate of scrub typhus were 15 and 0.1504 DALYs/100,000 in Linyi District, the initial foci of scrub typhus in Shandong Province. Populations of Linyi and Laiwu were 10,039,435 and 1,226,393, respectively. So, even though DALYs of scrub typhus was higher in Linyi than Laiwu, DALY rate was apparently higher in Laiwu than Linyin. Therefore, considering the limited health resources, more attention should be paid to Laiwu, a new focus of scrub typhus.

No cases of death were reported in Laiwu, China from 2006 to 2012, so the YLLs were 0 and the DALYs were equal to YLDs. There were several reasons why DALYs were adopted rather than solely YLDs. Worldwide acceptance of DALYs and DALY rate enabled international comparisons or ranking of areas by disease burden [8], [15], [16], [33]–[43]. DALYs represent unified summary estimate of disease burden that takes mortality as well as morbidity into consideration, i.e., DALYs and DALY rate would be more useful in comparing the disease burden among different diseases and different areas than other indexes. In addition, the levels of diagnosis and treatment of scrub typhus in Laiwu, China were high enough to cure all the patients, but there were still death cases in some other areas in China [44], [45]. For example, there were death cases of scrub typhus reported in Guangzhou, China in 2012. When comparing the disease burden of scrub typhus between these two districts, as a single metric DALYs is better than YLDs [44]. The DALYs of scrub typhus were equal to YLDs in Laiwu demonstrated that more attention should be paid to the unhealthy conditions of scrub typhus.

Since 2009, DALY rates and incidences of scrub typhus have both been increasing year by year (Table 3 and Table 4). With global warming, the predicted scenarios of increased temperature and rainfall were also causing concern for increases in vector-borne diseases, particularly, endemic arboviruses [46]. In 2010, Kim SH and Jang JY [47] reported that the incidence of scrub typhus in hyper-endemic region during the outbreak period was positively correlated with temperature and humidity during the summer. In addition, scrub typhus was transmitted to humans by the bites of chiggers which are mainly active in forest clearings, riverbanks, and grassy regions [47], [48]. With the increased urbanization and higher green coverage rate, people have more opportunity to contact chiggers. In 2012, there were several death cases of scrub typhus reported in Guangzhou, China and these patients had lied, stood, or sat on lawn in gardens before onset of the disease [44]. Green coverage rate achieved 42.19% and park green space per capita was 18.49 square meters in Laiwu, China in 2012 [49]. More suitable environment for chiggers' multiplication, more cases might appear in Laiwu.

From 15 to 80 years old, the average annual DALY rate and incidence of scrub typhus in females were both higher than males (Table 3, Table 4). Moreover, in the 60–69 years age group, females had a sharply higher DALY rate (2.6095 DALYs/100,000) than males (1.5689 DALYs/100,000) (Fig. 2). Thus, females, especially those from 60 to 69 years old, were the highest risk population for scrub typhus in Laiwu, China. As a zoonosis, scrub typhus was infected with *O. tsutsugamushi*, and rodents were the main host of *O. tsutsugamushi*. With increasing touch chance with *O. tsutsugamushi*, people in fields were easier to suffer from the disease [50]. Being a modern industrial area, Laiwu had become one home of numerous iron and steel businesses in China. Therefore, with more and more young and males moving from rural to urban areas, more and more elderly and females now engage in agriculture activity [18]. Additionally, with sparse awareness of the disease, the elderly have a greater chance of contact with chiggers [18]. Hence, health education efforts regards scrub typhus should be focused upon high risk groups like females who are 60–69 years old, especially those who live in countryside.

SARIMA modeling was useful for interpreting and applying surveillance data in disease control and prevention [51], [52]. As with many infectious diseases, the time series data of scrub typhus in Laiwu, China showed the components of trend and seasonal pattern. One of the most recognized disadvantages of this approach is the necessity of a large amount of data (i.e., a minimum of 50 observations) to build a reasonable SARIMA model [53]. In this research, monthly number of cases from 2006 to 2011 (72 observations) was used to build the SARIMA model, and monthly cases during the period of January to December in 2012 were used to validate the corresponding SARIMA model. The chosen SARIMA(2,1,0)(0,1,0)_12_ model fit the observations well and the residual series were satisfied with white noises. Therefore, the SARIMA(2,1,0)(0,1,0)_12_ model could be used to forecast the monthly cases of scrub typhus in Laiwu, China. The prevailing disease occurred in autumn and peaked in October, which suggests that education and other protective measures should occur just before October.

In the research, we adopted the incidence- and pathogen-based DALYs approach used by BcoDE Project to estimate the disease burden of scrub typhus rather than prevalence-based DALYs method presented in the GBD 2010. In case of an infectious pathogen, and in particular for priority settings of intervention to prevent primary infection, incidence is a more appropriate input for the DALYs metric than prevalence with the reason that only with the initial start of the infection is it possible to include all the disease squeals that result from the infection [12]. In addition, the incident cases of scrub typhus in Laiwu could be obtained from surveillance systems accurately. Considering above mentioned, the incidence- and pathogen-based DALYs were adopted in this research.

Limitations of this research should be acknowledged. The occurrence of scrub typhus was influenced by many factors such as pathogen prevalence, mites, rodents, human being's activities, and social or environmental factors. While building the SARIMA model, factors mentioned above were not considered. In addition, our research regarding disease burden and time series analysis of scrub typhus only focused on Laiwu, China. The results may not generalize to the most of China's population.

The disease burden of scrub typhus has increased year-by-year in Laiwu, China. Public health authorities should make concerted efforts to control and prevent the disease. The results of disease burden can also assist authorities in identifying the high-risk population. The SARIMA(2,1,0)(0,1,0)_12_ model developed in the research could offer prediction of scrub typhus monthly cases in Laiwu. Combined with disease burden measurement and SARIMA forecasting, these data should help official as a decision support tool in a scrub typhus risk management program and in planning various prevention efforts.
